# Exploring Older Adult Health Literacy in the Day-to-Day Management of Polypharmacy and Making Decisions About Deprescribing: A Mixed Methods Study

**DOI:** 10.3928/24748307-20221216-01

**Published:** 2023-01

**Authors:** Robyn Gillespie, Judy Mullan, Lindsey Harrison

## Abstract

**Background:**

Polypharmacy use in older adults is increasing and sometimes leads to poor health outcomes. The influence of health literacy in managing polypharmacy and making decisions about stopping medication has received limited attention.

**Objective:**

A mixed methods design was used to measure and investigate the influence of health literacy in the management of polypharmacy and decisions about deprescribing. Phase 1 involved two cross-sectional surveys, one with older adults using five or more medications and the other with general practitioners (GPs).

**Methods:**

Older adult health literacy was measured using the All Aspects of Health Literacy Scale. Phase 2 employed individual interviews with both older adults and GPs and further explored the reported use of health literacy in practice. SPSS version 24 was used to conduct descriptive statistical analysis of the Phase 1 survey responses and Phase 2 interviews were analyzed using thematic analysis with the assistance of NVivo 12.

**Key Results:**

Phase 1 survey responses were received from 85 GPs and 137 older adults. Phase 2 interviews were conducted with 16 GPs and 25 older adults. Phase 1 results indicated that self-reported older adult health literacy was high, and that GPs believed older patients could engage in decisions about deprescribing. Phase 2 findings showed that older adults developed and employed complex health literacy practices to manage medications between consultations; however, few reported using their health literacy skills in consultations with their GPs. GPs noted that older adult involvement in decision-making varied and generally thought that older adults had low health literacy.

**Conclusion:**

Older adults reported using health literacy practices in the management of their sometimes-complex medication regimens. However, the role of health literacy in deprescribing decision-making was limited. The mixed methods approach allowed greater insight into older adult and GP practices that influence the acquisition and use of health literacy. [***HLRP: Health Literacy Research and Practice*. 2023;7(1):e14–e25.**]

**Plain Language Summary:**

This report explores health literacy in the use of multiple medications and decisions to stop using medication/s in older age. Older adults reported good heath literacy and practiced many health literacy skills in the management of their medications. However, they did not always report the use of their health literacy skills when discussing their medications with their family doctor.

Polypharmacy, commonly described as the use of five or more medications, is increasingly prevalent, especially among older adults (World Health Organization, 2019). Polypharmacy can be beneficial when managing multiple morbidities (Duerden et al., 2013; Hughes et al., 2014; Lee et al., 2020). However, its use in older adults also poses a greater risk of medication-related harm (Hanlon et al., 2017; Mangin et al., 2018) and medication burden associated with increased personal costs, managing side effects, and managing complex medication regimens (Abu Dabrh et al., 2015; Linsky et al., 2019). For these reasons, deprescribing or stopping medications could be considered as a useful strategy to help withdraw inappropriate medication, manage polypharmacy, and improve outcomes (Reeve et al., 2015).

General practitioners (GPs) do most medication prescribing in the community setting (Britt et al., 2016), are trusted and can assist an older person to decide about whether to continue or stop a medication (Gerlach et al., 2020). However, variable participation by older adults in deprescribing decision-making with their GPs has been reported (Weir et al., 2017).

Many factors influence older adult participation in shared decision-making including limited time in consultations, attitudes of deference to medical professionals, and GP training (Bastiaens et al., 2007; Blumenthal-Barby, 2017). It is known that older adults face significant decision-making demands when considering the competing priorities of the benefits and potential (or current) side effects or burdens of polypharmacy use (Bokhof & Junius-Walker, 2016; Fried et al., 2008). Older adults may not have their medications routinely reviewed resulting in missed opportunities to participate in medication-related decisions (Frank, 2014). Commentary on deprescribing suggests that older adult decision-making preferences may be influenced by their health literacy levels, including their ability to comprehend health information and/or to communicate their medication preferences with their doctors (Holmes & Todd, 2017).

The risk of low health literacy is high in older adults and is associated with lower cognition and male gender (Clouston et al., 2017; Ganguli et al., 2021). Those with low health literacy are more likely to use an increased number of medications especially if they are male (Shebehe et al., 2022). A review of medication related burden indicated inadequate or sometimes conflicting medication information was given to older adults (Mohammed et al., 2016). The potential problems associated with this are likely to be compounded for those with poor health literacy in this group.

Nutbeam (2000) has defined health literacy as “the cognitive and social skills which determine the motivation and ability of individuals to gain access to, understand and use information in ways which promote and maintain good health.” Broad conceptualizations of health literacy suggest that rather than it being a static, measurable skill, health literacy is more usefully thought of as an asset that develops and changes over time, is distributed throughout a community and shapes context specific everyday practices (Edwards et al., 2012, 2015). Such conceptualizations focus on the practice of health literacy. However, most previous health literacy research has focused on measuring health literacy as an individual skill and have neglected using qualitative methods to capture health literacy practices (Pinheiro, 2021). In this study, we measured older adult health literacy and explored descriptions of the use of health literacy in daily life, specifically in the management of polypharmacy and in decisions about deprescribing. We also investigated GPs' descriptions of their interactions with their older patients regarding medication decision-making. We examined the usefulness of a mixed methods approach to the exploration of health literacy. This study is part of a larger body of work exploring the factors which influence deprescribing in primary care in Australia.

## Methods

The criteria from O'Cathain et al. (2008) for reporting mixed methods studies was used to guide this report.

### Study Design

Mixed methods research allows multiple types of evidence to be gathered and integrated resulting in a greater depth of knowledge (Johnson & Onwuegbuzie, 2004). Chinn and McCarthy (2013) recommend a mixed methods approach to explore health literacy as it allows the complexities that contribute to health literacy to be explored.

**Figure 1.**
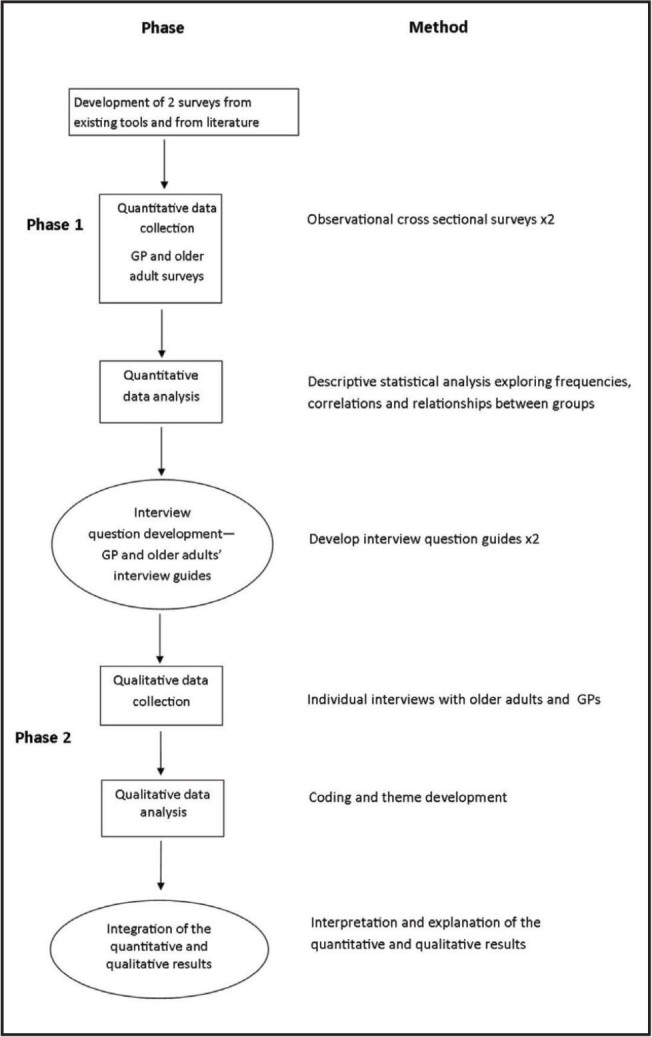
Study design. GP = general practitioners

This study used a sequential explanatory approach (Creswell & Clark, 2017). The findings of the initial quantitative phases were used to inform the development of focused question guides. This allowed the quantitative findings to be explored in more depth in the later qualitative phase. **Figure 1** describes the design of the study, which included GPs and older community living adults age 65 years or older who were taking five or more medications. Two phases were conducted, each with two study arms. In both study phases, older adults were included if they were living in the community and using five or more medications. There were no exclusion criteria applied to GPs.

In the first phase, two cross-sectional surveys were conducted, one with GPs and the other with older adults. Surveys were completed between October 2015 and November 2016. As no validated tool existed, a 21 item GP survey tool was developed to explore attitudes toward deprescribing. This was based on a review of the available deprescribing literature (Anthierens et al., 2010; Bell et al., 2015; Linsky et al., 2015; Moen et al., 2010; Schuling et al., 2012). The survey was piloted with five GPs to check usability and face validity. Further discussion about the development of the GP survey has been published (Gillespie et al., 2018).

The older adult survey included two validated tools: the Patients' Attitude Towards Deprescribing (PATD) questionnaire (Reeve et al., 2013) and the self-reported All Aspects of Health Literacy Scale (AAHLS) (Chinn & McCarthy, 2013). For this study, the three AAHLS questions, which address health literacy at the level of community engagement were not included because they were not relevant to the study objectives. In the absence of a scoring method for the AAHLS, at the time of this study, lower summed scores were assumed to indicate lower health literacy capabilities. The older adult survey also included three items from the Canadian Survey of Experiences with Primary Health Care that investigated recall of medication reviews, involvement in decisions about medications and perceptions of adequate time in consultations to discuss medication concerns (Statistics Canada, 2009). Demographic data were collected. A five-point Likert scale was used to measure quality of life (Krzych et al., 2018) and self-rated overall health. Postcode was used to derive the level of socioeconomic status for each participant, using SEIFA (Socio-Economic Indexes for Areas) score.

In the second phase, individual interviews were conducted with GPs and older adults. Separate interview question guides were developed for both GPs and older adults based on the analysis of the phase one results. Interviews, conducted between January 2018 and May 2019, were recorded with permission, transcribed verbatim, and pseudonyms were used to ensure participant anonymity.

### Study Setting, Sampling, and Ethical Considerations

The study was carried out in an area covering both urban and semi-rural areas south of Sydney, Australia. The demographic profile of the study area regarding the percentage of people older than age 65 years and provision of and utilization of GP services is similar to Australia's as a whole (Ghosh et al., 2013).

A convenience sample of both GPs and older adults was gathered for Phase 1. GPs were recruited via their practices while older adults were recruited via community pharmacies and seniors' community groups. In Phase 2, GPs were recruited via professional networks and snowballing. Older adults who had participated in the earlier survey and were willing to be interviewed were included and additional individuals were contact via snowballing. Sample size for the qualitative phase was not pre-determined. However, data collection ceased when saturation was achieved in both groups.

The study was approved by the University of Wollon-gong and Illawarra Shoalhaven Local Hospital District and the Medical Human Research Ethics Committee (HREC No: 15_086).

### Data Analysis

Descriptive statistical analysis was conducted using SPSS version 24 for the Phase 1 responses. Statistical significance was assumed where a *p* value reached ≤ .05.

Thematic analysis (Braun & Clarke, 2012) was used to analyze the Phase 2 qualitative data. GP and older adult transcripts were analyzed separately using an inductive approach assisted by NVivo 12 (QSR International Pty Ltd, 2018). One researcher (R.G.) carried out the initial coding, comparing codes across the data set. Codes were discussed among the team and agreement on major themes was reached in team meetings.

The final integrated analysis of Phase 1 and Phase 2 explored complementary, convergent, or divergent patterns or findings (Fetters & Molina-Azorin, 2019; Morgan, 2019). Both forms of data were considered equal, not elevating the findings of one over the other.

## Results

In Phase 1, 85 GPs and 187 older adults responded to their respective surveys. Fifty older adult responses were excluded because respondents did not meet the inclusion criteria for age and/or number of medications used. Data quality was high across both surveys with missing responses for individual items ranging from 0.0% to 5.1%.

The characteristics of the survey respondents are described in **Table 1** and**Table 2**. The median GP age was 52 years and 57% were male. Almost one-half (47.6%) were experienced practitioners. The median age of older adults was 76 years and 60.5% were female. They were taking a median of seven medications. Just more than one-half (51.1%) had completed a form of post-high school training. In phase 2, 16 GPs and 25 older adults were interviewed. Interviews ranged in length from 17 to 53 minutes.

**Table 1:**
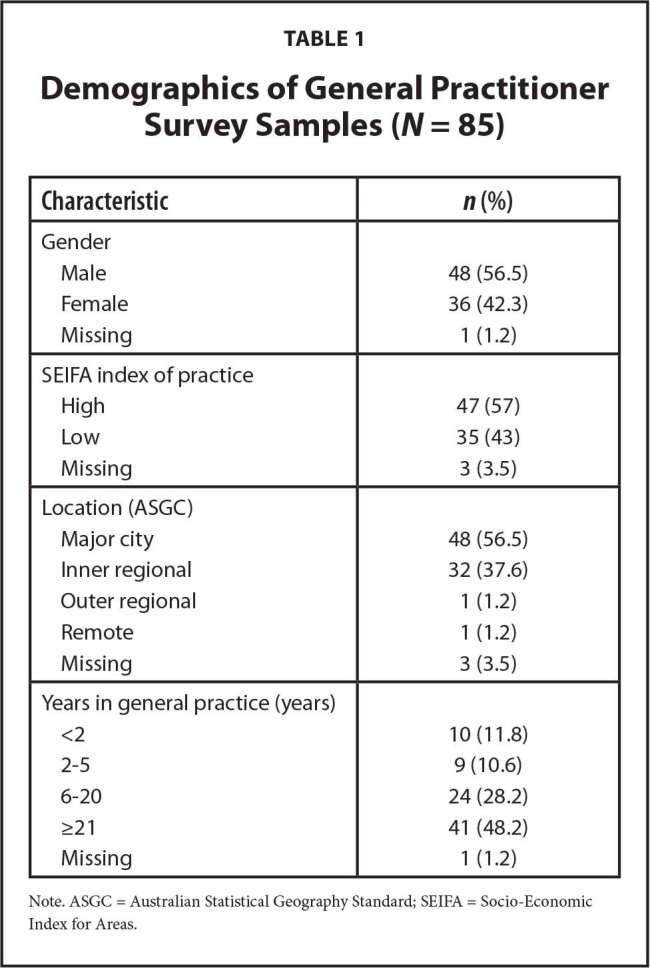
Demographics of General Practitioner Survey Samples (*N* = 85)

**Characteristic**	***n* (%)**

Gender	
Male	48 (56.5)
Female	36 (42.3)
Missing	1 (1.2)

SEIFA index of practice	
High	47 (57)
Low	35 (43)
Missing	3 (3.5)

Location (ASGC)	
Major city	48 (56.5)
Inner regional	32 (37.6)
Outer regional	1 (1.2)
Remote	1 (1.2)
Missing	3 (3.5)

Years in general practice (years)	
<2	10 (11.8)
2–5	9 (10.6)
6–20	24 (28.2)
≥21	41 (48.2)
Missing	1 (1.2)

Note. ASGC = Australian Statistical Geography Standard; SEIFA = Socio-Economic Index for Areas.

**Table 2:**
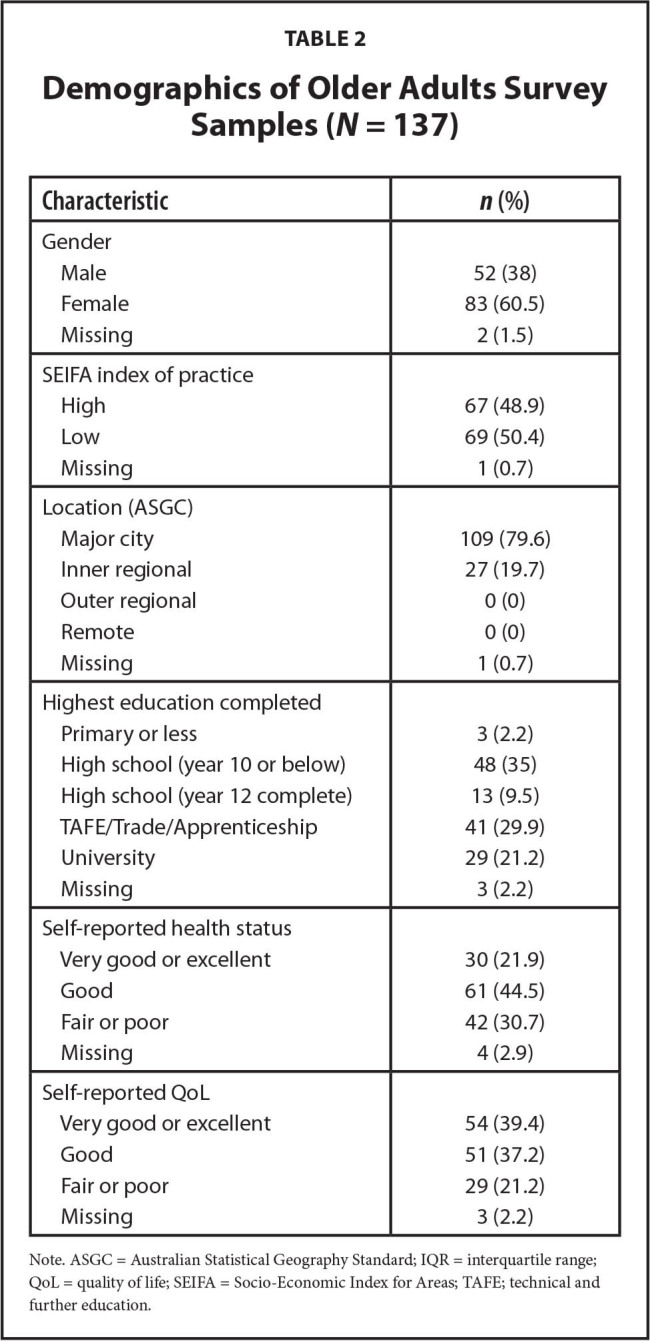
Demographics of Older Adults Survey Samples (*N* = 137)

**Characteristic**	***n* (%)**

Gender	
Male	52 (38)
Female	83 (60.5)
Missing	2 (1.5)

SEIFA index of practice	
High	67 (48.9)
Low	69 (50.4)
Missing	1 (0.7)

Location (ASGC)	
Major city	109 (79.6)
Inner regional	27 (19.7)
Outer regional	0 (0)
Remote	0 (0)
Missing	1 (0.7)

Highest education completed	
Primary or less	3 (2.2)
High school (year 10 or below)	48 (35)
High school (year 12 complete)	13 (9.5)
TAFE/Trade/Apprenticeship	41 (29.9)
University	29 (21.2)
Missing	3 (2.2)

Self-reported health status	
Very good or excellent	30 (21.9)
Good	61 (44.5)
Fair or poor	42 (30.7)
Missing	4 (2.9)

Self-reported QoL	
Very good or excellent	54 (39.4)
Good	51 (37.2)
Fair or poor	29 (21.2)
Missing	3 (2.2)

Note. ASGC = Australian Statistical Geography Standard; IQR = interquartile range; QoL = quality of life; SEIFA = Socio-Economic Index for Areas; TAFE; technical and further education.

### Phase 1

GP and older adult survey results reporting attitudes toward deprescribing have been reported elsewhere (Gillespie et al., 2018; Gillespie et al., 2019)

### GP Survey

Most GPs (84.7%) agreed that their older patients could engage in decision-making. Almost two-thirds agreed that they would ask patients their preference when deciding to continue or deprescribe a medication. GPs' views on their older patients' preferences for involvement in decisions varied with almost one-half agreeing that their older patients preferred to defer decision-making to them. Most GPs reported that they could identify when their older patients found it difficult to understand the rationale for deprescribing. This suggests that they were assessing, in a practical sense what could be described as the health literacy level of their older patients. Many (68.2%) GPs believed they had the ability to provide and communicate risk and benefit information about deprescribing with their older patients (**Table 3**).

**Table 3:**
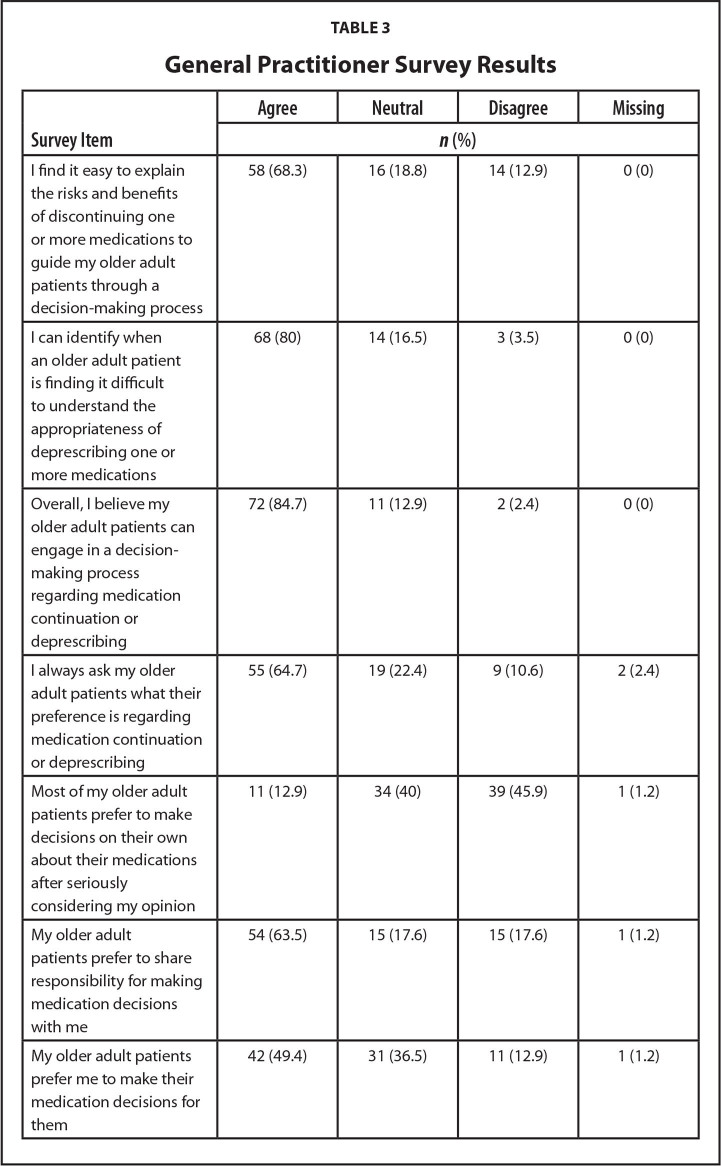
General Practitioner Survey Results

**Survey Item**	**Agree**	**Neutral**	**Disagree**	**Missing**
	***n* (%)**			
I find it easy to explain the risks and benefits of discontinuing one or more medications to guide my older adult patients through a decision-making process	58 (68.3)	16 (18.8)	14 (12.9)	0 (0)
I can identify when an older adult patient is finding it difficult to understand the appropriateness of deprescribing one or more medications	68 (80)	14 (16.5)	3 (3.5)	0 (0)
Overall, I believe my older adult patients can engage in a decision-making process regarding medication continuation or deprescribing	72 (84.7)	11 (12.9)	2 (2.4)	0 (0)
I always ask my older adult patients what their preference is regarding medication continuation or deprescribing	55 (64.7)	19 (22.4)	9 (10.6)	2 (2.4)
Most of my older adult patients prefer to make decisions on their own about their medications after seriously considering my opinion	11 (12.9)	34 (40)	39 (45.9)	1 (1.2)
My older adult patients prefer to share responsibility for making medication decisions with me	54 (63.5)	15 (17.6)	15 (17.6)	1 (1.2)
My older adult patients prefer me to make their medication decisions for them	42 (49.4)	31 (36.5)	11 (12.9)	1 (1.2)

### Older Adult Survey

Older adults generally reported high health literacy scores, especially on the functional (18–20) and communicative health literacy domain questions (19–23). Scores for the critical health literacy domain were more varied (24–27) (see **Table 4**). Respondents with lower overall AAHLS scores were significantly more likely to be in the group using 10 or more medications (Mann Whitney *U* = 921.50, *p* = .003).

**Table 4:**
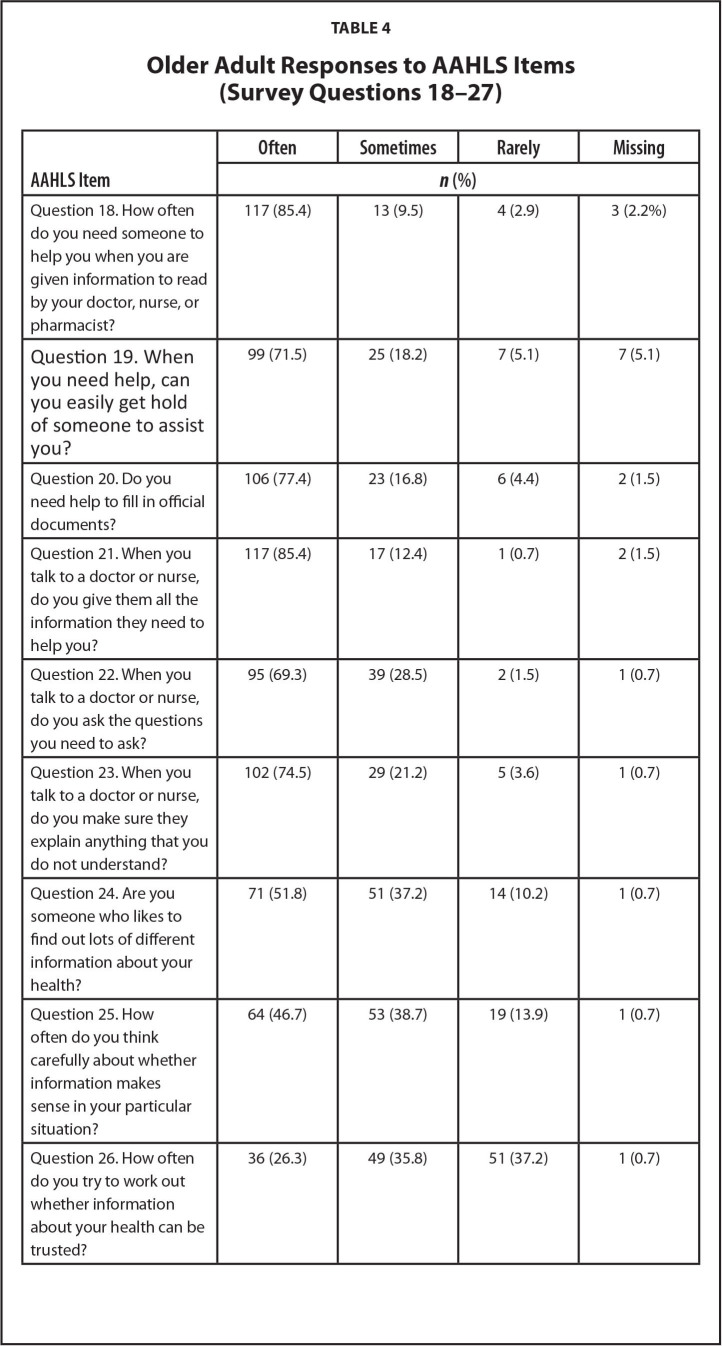

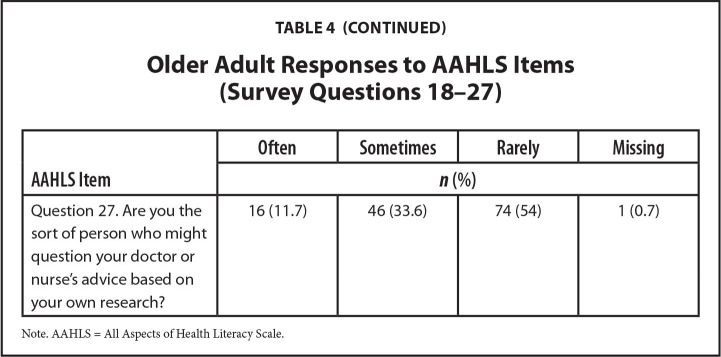
Older Adult Responses to AAHLS Items (Survey Questions 18–27)

**AAHLS Item **	**Often**	**Sometimes**	**Rarely**	**Missing**
	***n* (%)**			
Question 18. How often do you need someone to help you when you are given information to read by your doctor, nurse, or pharmacist?	117 (85.4)	13 (9.5)	4 (2.9)	3 (2.2%)
Question 19. When you need help, can you easily get hold of someone to assist you?	99 (71.5)	25 (18.2)	7 (5.1)	7 (5.1)
Question 20. Do you need help to fill in official documents?	106 (77.4)	23 (16.8)	6 (4.4)	2 (1.5)
Question 21. When you talk to a doctor or nurse, do you give them all the information they need to help you?	117 (85.4)	17 (12.4)	1 (0.7)	2 (1.5)
Question 22. When you talk to a doctor or nurse, do you ask the questions you need to ask?	95 (69.3)	39 (28.5)	2 (1.5)	1 (0.7)
Question 23. When you talk to a doctor or nurse, do you make sure they explain anything that you do not understand?	102 (74.5)	29 (21.2)	5 (3.6)	1 (0.7)
Question 24. Are you someone who likes to find out lots of different information about your health?	71 (51.8)	51 (37.2)	14 (10.2)	1 (0.7)
Question 25. How often do you think carefully about whether information makes sense in your particular situation?	64 (46.7)	53 (38.7)	19 (13.9)	1 (0.7)
Question 26. How often do you try to work out whether information about your health can be trusted?	36 (26.3)	49 (35.8)	51 (37.2)	1 (0.7)
Question 27. Are you the sort of person who might question your doctor or nurse's advice based on your own research?	16 (11.7)	46 (33.6)	74 (54)	1 (0.7)

Note. AAHLS = All Aspects of Health Literacy Scale.

### Relationship Between PATD Items and Summed Health Literacy Scores

The relationship between attitudes toward medication use or deprescribing and health literacy was explored. The findings indicate that willingness to consider stopping medication(s) correlated with higher scores on the critical health literacy subscale (*rs* = .198, *p* < .021) and the summed overall scores (*rs* = .229, *p* < .009). In contrast, there was a negative correlation between both functional (rs = −.244, *p* < .005) and communicative health literacy scores (rs = −.266, *p* < .002) and feeling that one or more medications may no longer be needed. Significant correlations were weak to moderate (Dancey & Reidy, 2007).

It is notable that higher overall AAHLS scores were positively correlated with understanding the reasons for medications being prescribed, participating in the decision-making process, recalling a medication review, and believing there was enough time to discuss medication concerns during consultations (**Table 5**). All of these factors are likely to be supportive of older adults' engaging with their health care provider to discuss deprescribing as a possible medication management strategy.

**Table 5:**
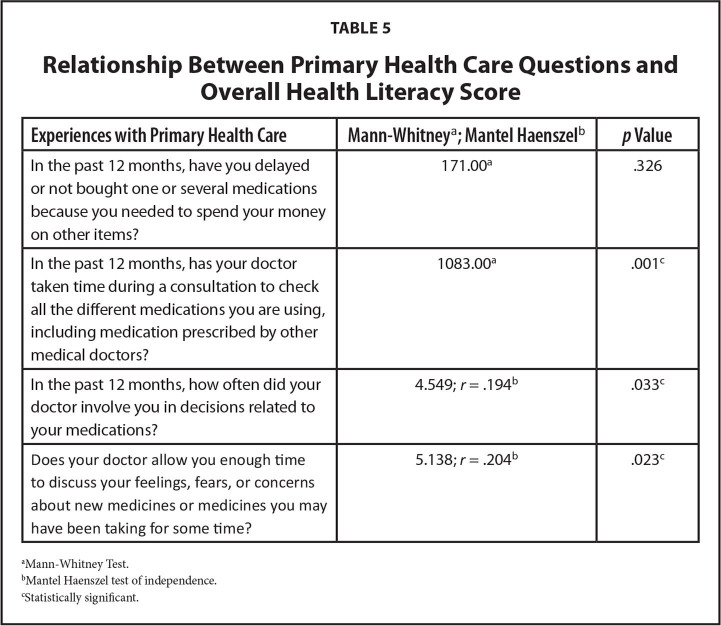
Relationship Between Primary Health Care Questions and Overall Health Literacy Score

**Experiences with Primary Health Care**	**Mann-Whitney** ^a^ **; Mantel Haenszel** ^b^	***p* Value**
In the past 12 months, have you delayed or not bought one or several medications because you needed to spend your money on other items?	171.00^a^	.326
In the past 12 months, has your doctor taken time during a consultation to check all the different medications you are using, including medication prescribed by other medical doctors?	1083.00^a^	.001^c^
In the past 12 months, how often did your doctor involve you in decisions related to your medications?	4.549; *r* = .194^b^	.033^c^
Does your doctor allow you enough time to discuss your feelings, fears, or concerns about new medicines or medicines you may have been taking for some time?	5.138; *r* = .204^b^	.023^c^

a Mann-Whitney Test.

b Mantel Haenszel test of independence.

c Statistically significant.

### Phase 2 Results

In phase 2, 16 GPs and 25 older adults participated. Most GPs (*n* = 9) had worked for more than 21 years. The majority were male (*n* = 10) and worked in low socioeconomic areas (*n* = 10). The age range of the older adults was 67 to 95 years with a median age of 79. Most participants used 5 to 9 medications (*n* = 16), and 14 lived in a low socioeconomic area.

### GP Individual Interviews

The results from the GP individual interviews gave further insight into the GPs' attitudes toward their older patients' health literacy capacity, suggesting a more varied attitude compared to the survey responses. Four themes were identified: doubt about older adult health literacy, strategies used to assist with poor health literacy, strategies to support shared decision-making, and recognition of health literacy demands on older adults.

Most GPs expressed or implied concern about their older patients' health literacy across the range of health literacy domains: functional, communicative, and critical. They noted their older patients' difficulty in following instructions, difficulty in understanding risk versus benefit and lack of ability to discern appropriate medication information. This led some to be cautious about deprescribing because of concerns that medication changes would result in confusion.

- “Getting confused. Yeah. Because you're taking away medications that have been there for a long time.” –GP 9
  Participants suggested various strategies to accommodate their patients' limited health literacy capabilities. For instance, they provided medication instructions slowly and/or repeatedly, as well as by providing instructions to relatives or caregivers. Involvement of older patients in decisions was limited. They held a pessimistic view of the potential usefulness of trying to improve their older patients' understanding.
- “I mean you can always say you want to educate patients but health literacy depends on each individual patient and no matter what you do, there's always going to be some patients that just don't get it.” –GP 5
  In contrast, a small group of participants expressed more positive views of their older patients' ability to participate in shared decision-making. They described practices they employed to support shared decision-making such as explaining the risks versus benefits of ongoing medication. They engaged their older patients in decisions, including about deprescribing, taking time to hear and respect their patients' preferences. This included exploring potential fears such as reoccurrence of symptoms or occurrence of preventable events following deprescribing. They sought to encourage their older patients to ask questions, such as “do I really need to take all these pills?” (GP 8).
  The health literacy demands placed on older adults were recognized by some GPs. For example, they acknowledged that generic medications often caused confusion and that medication information was limited when using blister packaged medications because the packs only included the name and dose of the medication. Also, patients did not always have medication changes explained to them during hospitalizations or specialist consultations.
- “I would imagine most people can juggle you know two or three medications reasonably easily but, obviously the more you have, the harder it potentially becomes and the more room for error there is.” –GP 13

### Older Adult Individual Interviews

Three themes were identified when analyzing the older adult qualitative data. These included: information gathering about medications, the use of health literacy skills in everyday medication management, and health system barriers to health literacy accumulation and application.

***Information gathering about medications.*** Finding information was a significant task undertaken by older adult participants. This information increased medication-related health literacy. A range of sources were accessed including their GP, specialists, pharmacists, practice nurses, patient information leaflets, the internet and medication information available from health magazines and books, as well as from family and friends. They also gathered their own autobiographical information regarding the history of their medication use, what benefits they experienced, problems or side effects.

The types of information sought included details about why each medication was required and how each medication worked and its potential side effects, including interactions between other medications. Participants also accessed information about administration such as when or how to take their medication, how best to store it and consequences associated with missing or changing dosages or changing medication types.

Apart from their GP, the internet was the most common source of medication information accessed. This was especially notable given their median age (76 years) and that GPs did not believe their older patients were accessing the internet. Some demonstrated the use of critical health literacy skills to assess the credibility, suitability, and usability of information to their health context. One of the outcomes of this increased knowledge was the ability to engage with their doctors, using their understanding of their own information needs to frame further questions during consultations.

- “Well. . .you pick up Google, don't you? And you look for reliable—as far as you know—thing and read it. But it gives me the basis for asking a question if I wanted a question to ask. Not believing everything they say by any means but giving me a bit more understanding.” –Participant, age 87 years
  Despite demonstrating significant knowledge of their own medications, older adults understood their GPs held specific medical knowledge and expertise and valued this more than their own. This view sometimes undermined their confidence to share their own knowledge and most chose to remain passive and trusting in decision-making.
- “Well, I read up on things. I've got one or two medical books. . .I do not ask him if I should be stopping any minute. I trust that he knows best because I'm not a medical man, am I?” –Participant, age 86 years

***Applying health literacy to manage medications.*** The participants noted the application of their health literacy skills in the many activities that they undertook to manage their medications daily. These included keeping their medications organized, getting prescriptions, collecting supplies, and setting reminders to take medications on time (often scheduling multiple doses per day and often around other activities). Additionally, participants actively monitored the effect of their medication use, including observing for side effects, improvements in their symptoms or outcomes following deprescribing, and communicated information back to their GPs. Because of their many medications and the frequent changes to regimens this was not an insignificant task. However, this monitoring and reporting back was important as it enabled their prescribers to find an acceptable balance of benefit versus side effects and had the potential to facilitate shared decision-making.

Participants also indicated that they actively weighed up the current usefulness of their medications as a resource to advance their goals to maintain their health, independence and to help extend their lives, against their assessment of risk in terms of current or future side effects.

- “It depends on the extent of the side effects. I mean you have to decide whether it was better to change”. . .“cause the side effects may be just as fatal as the medicine sort of thing.” –Participant, age 88 years

***Health system barriers to the accumulation and use of health literacy.*** The development of older adult health literacy skills was not always supported during consultations. Older adults reported not always being told relevant information about their medications or leaving consultations unsure of the meaning of explanations. They noted they did not always ask the questions they needed to about their medications because they trusted the expertise of their GP and specialists. Some did not believe that their GP had enough time to provide them with adequate information about medications. Those participants who were frail were no longer able to access their GP independently, resulting in less frequent access to their GP as a source of medication information.

- “I think they're time-poor. They're pushing you through, so you really don't get very much information. And they're the doctor . . . but I always do my own checking up.” –Participant, age 67 years
  The participants' experience of medication reviews varied. Only six described regular, comprehensive reviews, where they were offered opportunities to discuss their medications. Others were unsure if their medications had ever been comprehensively reviewed and had limited opportunities to share their treatment preferences to inform decision-making.
- “We talk about the medications . . . she sort of runs through them all, but she doesn't necessarily talk about them all, you know what I mean? I think she just sort of seems to accept that if things are going okay that we'll stick with that.” –Participant, age 88 years

## Discussion

This study measured older adult health literacy and explored its use in the management of polypharmacy and decision-making about deprescribing. Self-reported health literacy was high, especially functional and communicative health literacy. The distribution of scores across the three domains was similar to that seen in a study of chronically ill patients conducted in the Netherlands using the Functional Communicative and Critical Health Literacy Scale (Heijmans et al., 2015).

The subsequent qualitative Phase added further details about the type and sources of information accessed by older adults to inform their medication related health literacy. Despite older adults demonstrating significant medication related knowledge and practicing complex health literacy skills between consultations they were less likely to report using their health literacy skills in consultations and GPs generally doubted the health literacy capability of their older patients.

Previously, the association between health literacy and older adult attitudes to polypharmacy use or deprescribing had been investigated finding no association (Schiøtz et al., 2018). Schiøtz et al. (2018) used a 4-item health literacy measure. However, using the more comprehensive AAHLS, higher health literacy scores were found to be associated with key aspects of polypharmacy management and attitudes toward deprescribing such as involvement in decision making, knowledge of medications and willingness to stop medication(s).

Older adults demonstrated that they had built their health literacy capacity over time and from a range of professional and lay sources, including the internet. This finding supports broader conceptions of health literacy which acknowledge that health literacy is co-produced within social relations (Samerski, 2019) and that health literacy is dynamic, as described in the model of distributive health literacy (Edwards et al., 2012, Edwards et al., 2015).

Earlier deprescribing studies suggested that GPs believed their older adults had a poor understanding of their medications due to old age or a lack of education (Linsky et al., 2015; Schuling et al., 2012). However, this study demonstrated that older adults' understanding about their medications was limited by missing information, a lack of time in consultations and variable involvement in medication reviews. This finding suggests that GPs should not assume that their patients have all the relevant information about their medications to enable them to participate in decision-making. Additional steps are required to ensure their patients are adequately informed about the indication, purpose, duration of treatment, and ongoing benefits/risks of their medication(s). Medication reviews are one opportunity to provide this information.

Despite demonstrating considerable knowledge of their own medications, many older adults trusted their GP's greater expertise and remained passive in consultations. Others demonstrated that their knowledge allowed them to be more empowered to enter discussions with their GP about their medications. Differences in critical health literacy capabilities may explain variability in involvement. GPs may interpret that older adults who are passive in consultations are lacking in health literacy skills. This, along with a general perception that older adults have deficient health literacy skills, may deter them from initiating shared decision-making, instead assuming decision-making authority.

Exploring and explaining decisions are opportunities to improve older adults' acquisition of health literacy capacity. Most GPs reported that they were confident to explain risks and benefits of medication use and deprescribing to their older patients. However, qualitative studies conducted elsewhere have found that GPs are uncertain of the risks/benefits of ongoing use of medications, especially in the case of preventatives, such as statins and they find it difficult to communicate risk/benefit information to their older patients (Anderson et al., 2017; Moen et al., 2010; Schuling et al., 2012; Wallis et al., 2017). Further investigation is required to understand what further support GPs need to enable them to support older adult decision-making.

This study has demonstrated the usefulness of a mixed method approach to exploring health literacy. Viewed in isolation, the quantitative analysis would have suggested a much more positive view of the association of good health literacy with aspects likely to be supportive of deprescribing such as participation in shared decision-making. However, the qualitative analysis allowed insight into the practical application of health literacy, revealing many barriers to its application when considering deprescribing and polypharmacy use.

## Study Limitations

The GP survey tool has not yet been validated and this may have affected the reliability of the findings in the initial GP quantitative arm. However, the GP survey results were also considered in light of the later findings from the qualitative arm and situated in relation to the current deprescribing literature. The samples across the quantitative arms of the study were small and based on convenience samples only. Most participants lived or practiced in an urban area, and it is not known if the results would be similar in rural or remote areas of Australia.

## Further Research

Only two participants in this study appeared to be in a frail condition and their health literacy practices were constrained. Demands on health literacy skills are likely to increase as frailty increases because treatments become more complex and varied and the outcomes of decisions are less certain. Further research is required with a larger sample to understand how best to support older adults who are in a frail condition to maintain and apply their health literacy skills and to support GPs as they seek to support shared decision-making when considering deprescribing.

Patient education materials are available to assist older adults as they consider deprescribing; however, the majority have been found to be difficult for average readers to comprehend and they do not present information in a way that would be sufficient to inform consent (Fajardo et al., 2019). This presents an opportunity for further improvement.

## Implications for Practice

Appropriate written and verbal communications about medications and providing information on trusted websites are important to empower all older adults to understand and access the information they require to participate in medication decisions. Involving older adults in regular reviews of the ongoing appropriateness of current medications may allow opportunities to discuss and plan deprescribing pro-actively without the time pressures of regular consultations.

## Conclusion

Significant demands are placed on the health literacy capabilities of older adults who use polypharmacy. A range of complex health literacy practices were used between consultations to independently manage their medications. However, health literacy was found to play a limited role in medication-related decisions. GPs were largely unaware of the extent of their older patient's health literacy capacity. Few opportunities were described to develop and to apply health literacy capabilities during discussions of polypharmacy use and/or deprescribing in consultations. These findings emphasize that GPs can do more to understand their older patient's health literacy capacity, refrain from assuming poor health literacy, and explore unmet information needs in the context of deprescribing.
